# *Notes from the Field:* Testing for Nonprescribed Fentanyl and Percentage of Positive Test Results Among Patients with Opioid Use Disorder — United States, 2019–2020

**DOI:** 10.15585/mmwr.mm7047a4

**Published:** 2021-11-26

**Authors:** Justin K. Niles, Jeffrey Gudin, Alana M. Vivolo-Kantor, R. Matthew Gladden, Desiree Mustaquim, Puja Seth, Harvey W. Kaufman

**Affiliations:** ^1^Quest Diagnostics, Secaucus, New Jersey; ^2^Department of Anesthesiology, Pain Management, and Perioperative Medicine, Miller School of Medicine, University of Miami, Miami, Florida; ^3^Division of Overdose Prevention, National Center for Injury Prevention and Control, CDC.

Overdose deaths involving synthetic opioids excluding methadone (primarily illicitly manufactured fentanyl) have increased approximately tenfold since 2013 (*1*) and have accelerated during the COVID-19 pandemic, with provisional estimates indicating that synthetic opioid–involved deaths increased 49.4% for the 12-month period ending April 2021.* During the pandemic, persons requiring medication for opioid use disorder (MOUD) might face additional challenges to accessing treatment (e.g., due to closure of providers’ offices) (*2*). Early in the pandemic, urine drug testing results indicated increases in nonprescribed fentanyl use (*3*,*4*). To determine trends in testing for fentanyl and the percentage of positive test results before and during the pandemic, clinical drug monitoring of urine specimens from patients residing in all U.S. states and the District of Columbia were tested for fentanyl by using definitive mass spectrometry at Quest Diagnostics during 2019–2020. A positive test result for nonprescribed fentanyl was defined as detection of norfentanyl (major fentanyl metabolite) or fentanyl not listed as prescribed.^†^ Patients receiving MOUD were identified as those having an *International Classification of Diseases, Tenth Revision, Clinical Modification* (ICD-10-CM) F11 code (opioid-related disorders)^§^ and a positive test result for buprenorphine or methadone listed as prescribed. Among 427,915 specimens, 53,969 (12.6%) from patients whose opioid use disorder medication status was inconclusive were excluded from the analyses.^¶^ Among the 373,946 included specimens, 57,749 (15.4%) were from patients receiving MOUD. SAS Studio (version 3.6; SAS Institute) was used to conduct all analyses. This activity was reviewed by CDC and was conducted consistent with applicable federal law and CDC policy.**

The numbers of specimens tested among patients receiving and not receiving MOUD declined 65% and 72%, respectively, during March 29–April 11, 2020 (weeks 14–15), compared with the same weeks in 2019. During September–December 2020 (weeks 35–52), the numbers of specimens tested among patients receiving and not receiving MOUD were 43% and 13% lower, respectively, compared with the same period in 2019.

In the first 2 full weeks of January 2019, before the beginning of the COVID-19 pandemic, 9.6% of specimens from patients receiving MOUD tested positive for nonprescribed fentanyl; the percentage testing positive approximately doubled by the end of 2019 (weeks 51–52) to 26.7% (Figure). During 2020, positive test result rates were highest during March 29–April 11 (weeks 13–14) when the percentage of positive test results peaked at 40.5%. Despite the decline in volume during this period, the overall demographic proportions of patients receiving drug testing remained similar (*3*), suggesting that substantial shifts in the patient demographics were not driving the increase. During April 26–May 9, 2020 (weeks 17–18), the percentage of positive test results declined to 24.3% and continued to decline during September–December 2020 (weeks 35–52; range = 11.9%–18.5% positive per week), levels considerably lower than the corresponding period during 2019 (range = 18.5%–26.7% per week). Among patients not receiving MOUD, the prevalence of positive nonprescribed fentanyl test results did not increase significantly during the early pandemic months compared with that in 2019 (Figure). A limited but significant increase during the second half of 2020 (weeks 27–52; range = 1.4%–1.8%) was observed, compared with the corresponding period during 2019 (range = 1.1%–1.7%).

A decline in drug monitoring disproportionately affected patients receiving MOUD during March–May 2020 (*3*) and continued through the end of 2020, raising concerns regarding potential treatment disruptions or patients forgoing monitoring tests during the pandemic. Naloxone prescriptions also disproportionately declined compared with all other medications during the pandemic and have remained at lower levels (*5*). These observations highlight the urgency of continuing and expanding access to MOUD and other treatment and harm reduction services, including, when indicated,^††^ resuming drug monitoring. In the context of predicted increases in opioid-involved and synthetic opioid–involved overdoses throughout 2020 (*6*), the continued lower test volume and lower percentage of positive test results for nonprescribed fentanyl during September–December 2020 among patients receiving MOUD should be investigated to ensure that patients at highest risk for health harms are receiving health care and are retained in care. Reported reductions in specimen submissions from New England and the Midwest (areas with higher nonprescribed fentanyl positivity rates) might also partially explain the lower percentage of positive nonprescribed fentanyl test results (*3*). With continued increases in the percentage of positive nonprescribed fentanyl test results among persons not receiving MOUD, intensified prevention of nonprescribed fentanyl use and overdose is urgently needed (e.g., fentanyl test strip dissemination, enhanced linkage to care, and expanded use of MOUD).^§§^^,^^¶¶^

**FIGURE:**
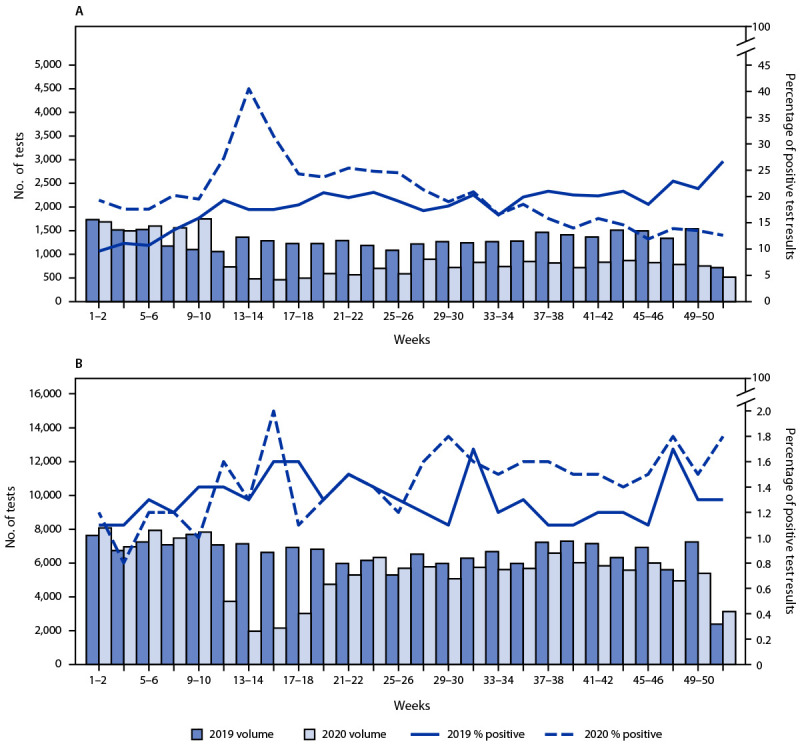
Biweekly nonprescribed fentanyl specimens tested and percentage of positive test results* among patients receiving (A)^†^ and not receiving (B)^§^ medication for opioid use disorder — United States, 2019–2020 **Abbreviation**: ICD-10-CM =* International Classification of Diseases, Tenth Revision, Clinical Modification.* * Primary and secondary y-axis scales are different between panels. ^†^ Patients receiving medication for opioid use disorder were defined as those with an ICD-10-CM code F11 (opioid related disorders) and a positive urine drug test result for buprenorphine or methadone indicated as prescribed. ^§^ Patients not receiving medication for opioid use disorder had no ICD-10-CM F11 code and a negative urine drug test result for buprenorphine and methadone.

## Footnotes

* https://www.cdc.gov/nchs/nvss/vsrr/drug-overdose-data.htm (Accessed July 15, 2021).

† Quest Diagnostics medMATCH uses clinician-provided prescribed medications (frequently assessed by using state prescription drug monitoring programs) to assess nonprescribed positivity. https://www.questdrugmonitoring.com/medmatch-reports

§ The ICD-10-CM F11 codes are administrative codes used to indicate a diagnosis from a physician and for billing purposes related to opioid abuse (F11.1), dependence (F11.2), or use (F11.9).

¶ Inconclusive MOUD status was assigned for positive test results for buprenorphine or methadone without an ICD-10-CM F11 code, ICD-10-CM F11 codes without buprenorphine or methadone positivity, or buprenorphine or methadone positivity not listed as prescribed.

** 45 C.F.R. part 46, 21 C.F.R. part 56; 42 U.S.C. Sect. 241(d); 5 U.S.C. Sect. 552a; 44 U.S.C. Sect. 3501 et seq.

††
https://www.asam.org/docs/default-source/quality-science/the-asam-appropriate-use-of-drug-testing-in-clinical-addiction-medicine-full-document.pdf?Status%20=%20Temp&sfvrsn%20=%20700a7bc2_2

§§
https://www.samhsa.gov/newsroom/press-announcements/202104070200

¶¶
https://emergency.cdc.gov/han/2020/han00438.asp
